# Editorial: RNA Localization and Localized Translation in Neurons

**DOI:** 10.3389/fnint.2021.831038

**Published:** 2022-01-12

**Authors:** Shannon Farris, Ezgi Hacisuleyman, Paul Donlin-Asp, Jean-Michel Cioni

**Affiliations:** ^1^Center for Neurobiology Research, Fralin Biomedical Research Institute at Virginia Tech Carilion, Roanoke, VA, United States; ^2^Department of Biomedical Sciences and Pathobiology, Virginia-Maryland College of Veterinary Medicine, Virginia Tech, Blacksburg, VA, United States; ^3^Laboratory of Molecular Neuro-Oncology, The Rockefeller University, New York, NY, United States; ^4^Max Planck Institute for Brain Research, Frankfurt am Main, Germany; ^5^Division of Neuroscience, Istituto di Ricovero e Cura a Carattere Scientifico (IRCCS) San Raffaele Scientific Institute, Milan, Italy

**Keywords:** RNA localization, local translation, RNA transport, neuron, axon, dendrite

Significant progress has been made in our understanding of how RNAs are regulated to provide spatial and temporal control over gene expression. However, due to the cell-type diversity and specialized morphology and physiology of neurons, much remains to be uncovered about how local RNA regulation and translation influence neuronal function. In this collection of reviews focused on “RNA Localization and Localized Translation in Neurons,” several aspects of RNA metabolism that are critical for localized RNA regulation are discussed. From transcription to transport and compartment-specific regulation, these reviews highlight the many knowns and unknowns of how local RNA regulation contributes to neuron function, how it is dysregulated in neurological diseases, and how emerging tools and technologies are revolutionizing our understanding of the functional roles local RNA regulation plays in neurons.

In their review, Agrawal and Welshhans (Front. Mol. Neurosci., 2021) discuss key findings from multiple studies on how distinct pools of RNAs localize in radial glial cells and axons during neuronal development. The active transport of RNAs bound by RNA binding proteins (RBPs) and their local translation have functional consequences for both, with roles in neurogenesis, differentiation, branching, synaptogenesis, and maintenance. The local transcriptome and the associated local translatome are modified and rendered more complex throughout development to meet the subcellular functional demands. Local translation in axons allows for the precise spatiotemporal regulation of gene expression by coordinating RNAs that are functionally related, especially in response to external stimuli. However, how this co-regulation is mediated mechanistically is still not well-understood. A handful of studies show that local proteomes can be remodeled by receptor interactions with local translational machinery, activation of signaling cascades that modify RBP-RNA interactions and/or the local translational machinery, or localized translation on specific organelles (e.g., endosomes). A better understanding of these molecular processes is becoming crucial as molecular dissection of the clinical phenotypes of neurodevelopmental diseases, such as Fragile X Syndrome, Autism Spectrum Disorders, and Down Syndrome, points to dysfunctional local translation in axons of cell adhesion molecules, transcription factors, translational machinery, and cytoskeletal- and membrane-associated proteins.

RBPs are key players in the regulation of RNA transport and local translation. Gamarra et al. highlight the importance of these proteins in a review discussing how mutations or depletions of RBPs can lead to a number of neurological and neurodegenerative disorders, including Amyotrophic Lateral Sclerosis and Parkinson's Disease. The molecular consequences of these mutations or depletions are discussed in depth, with a particular focus on how dysregulation of local translation may underlie and contribute to the development and progression of nervous system pathologies. What remains to be directly tested is whether these molecular changes in response to RBP mutations or loss drive neurological diseases through dysregulation of the local transcriptome or if these are secondary to other cellular adaptations. Further technological innovations will be needed to dissect out such mechanisms, for instance methods which would allow us to specifically target and manipulate RNAs in distinct neuronal compartments.

It has been reported that thousands of RNAs are enriched in neuronal compartments, and specific transcriptomic signatures have been observed in distinct subdomains (e.g., axon, dendrite, growth cone, synapse). Moreover, restricted pools of RNAs can also be recruited to these subcompartments in response to stimuli. In view of the morphological complexity of neuronal cells, one of the biggest challenges in the field is now to understand how RNA transport is controlled in a precise manner. In their review, Rodrigues et al., provide a comprehensive overview of “*the few knowns and many unknowns”* regarding the molecular principles underlying neuronal RNA trafficking. They describe in detail the direct and indirect evidence that have led to the discovery of many proteins involved in RNA transport. They highlight the specific roles of RBPs, adaptor/motor proteins and organelles in RNA distribution in axons and dendrites and describe the technical advances that are now opening the possibility to better decipher the essential components of the RNA trafficking machinery.

Advancements in RNA sequencing technologies and methods for cell-specific profiling have led to the identification of diverse cell- and compartment-specific transcriptomes. Contributing to this diversity is the extent of alternative isoform expression within and across neuronal subtypes. By changing the cis regulatory sequences of a given transcript, and thereby the associated trans acting RNA binding proteins and other interactors, many aspects of RNA metabolism—from transport to translation and degradation—can be impacted. The review by Park and Farris highlights the role of alternative isoform expression as one means of diversifying RNA regulation to provide spatial and temporal control over compartmentalized gene expression. They review several specific gene examples to illustrate how alternative isoform expression is used in hippocampal neurons to provide distinct subcellular compartments with the necessary transcripts to support specialized functions, with a focus on development and plasticity. They emphasize how unraveling this RNA regulatory code is vital to fully understanding how RNA is regulated across different contexts and cell types to support neuronal functions.

Technical hurdles have impeded our understanding of the functional role for localization of mRNA and protein synthesis. Minehart and Speer discuss state-of-the-art approaches being employed to understand these processes with subcellular resolution. These include methods from subcellular transcriptomics, translatomics, proteomics, and super-resolution imaging. In particular they highlight three critical areas of exploration: (1) molecular-genetic approaches for quantifying mRNA diversity, abundance, and regulation of the local transcriptome/translatome in targeted subcellular compartments, (2) proteomic techniques for assessing nascent proteins interaction networks in synaptic connections, and (3) super-resolution fluorescence microscopy for quantitative analysis of subsynaptic resolution in circuits. These approaches are currently revolutionizing our understanding of where mRNAs localize and the functional role their compartmentalization plays in neuronal function.

Multiple layers of RNA regulation highlighted in these reviews point to complex post-transcriptional and translational mechanisms that orchestrate neuronal function. Given the complexity of local RNA regulation in neurons, there are still numerous unanswered questions. Many lines of work in the field are geared toward understanding how neuronal development and different patterns and forms of neuronal activity can modify subcellular RNA metabolism and, in turn, how local changes in RNA metabolism influence neuronal activity and behavior. Neurons integrate a multitude of signals received over short and long distances and time scales. It remains unclear how different RNA regulatory mechanisms, such as splicing, localization, or local translation, play a role in (re)structuring proteomic states as a consequence of this complex signal integration. Another area of great interest lies in the transport of RNAs to distal sites. What is the subset of RNAs that travel to neurites and how is this transport mediated, particularly in response to changes in neuronal activity? What is the composition of RBPs and RNAs in transport granules across neuronal compartments and under different physiological states? How do the principles of RNA regulation differ across neuronal compartments and under different physiological states? Finally, the dynamics of protein synthesis and degradation is a topic of exciting investigation. How are the transcriptome and proteome remodeled in neurites throughout development and in response to plasticity and does this differ across cell types and conditions?

## Author Contributions

All authors listed have made a substantial, direct, and intellectual contribution to the work and approved it for publication.

## Conflict of Interest

The authors declare that the research was conducted in the absence of any commercial or financial relationships that could be construed as a potential conflict of interest.

## Publisher's Note

All claims expressed in this article are solely those of the authors and do not necessarily represent those of their affiliated organizations, or those of the publisher, the editors and the reviewers. Any product that may be evaluated in this article, or claim that may be made by its manufacturer, is not guaranteed or endorsed by the publisher.

